# Patient experiences of a 10-weeks weight-neutral treatment program for clinical binge eating disorder in a higher weight population

**DOI:** 10.1186/s40337-023-00955-0

**Published:** 2023-12-18

**Authors:** Kjersti Hognes Berg, Eli Natvik, Trine Tetlie Eik-Nes

**Affiliations:** 1 Department of Neuromedicine and Movement Science, Faculty of Medicine and Health Sciences, Norwegian University of Science and Technology, Trondheim, Norway; 2 Stjørdal Community Mental Health Centre, Nord-Trøndelag Hospital Trust, Levanger, Norway; 3 Department of Health and Caring Sciences, Faculty of Health and Social Sciences, Western Norway University of Applied Sciences, Førde, Norway; 4 The centre for Health Research, District General Hospital of Førde, Førde, Norway

**Keywords:** Stigma, Shame, Binge eating disorder, Higher weight, Obesity, Qualitative research

## Abstract

**Background:**

Weight based stigma might drive the development of both higher weight and binge eating disorder (BED). To improve treatment and outcomes, a deeper understanding of how stigma and shame are correlated in clinical encounters is needed. The current study was designed to gain insight into how participating in a 10-weeks weight-neutral treatment program for patients with binge eating disorder and higher weight was experienced.

**Methods:**

Semi-structured interviews were conducted with 10 patients who had completed the BED treatment. The intervention was group based, addressing stigma and shame, using models of attachment and affect regulation in the presentation of BED. Interviews were analyzed guided by van Manen’s hermeneutic-phenomenological approach.

**Results:**

A profound feeling of inferiority due to weight stigma and adverse childhood experiences appeared to have kept the participants stuck in a shame driven carousel  of dieting, weight loss, bingeing, and weight regain. Participants and health care professionals’ mutual acknowledgement of driving elements of binge eating appeared to support participants feeling more equal. Feeling equal was described as facilitating increased awareness and tolerance of bodily sensations and emotions, and a deeper understanding and self-caring attitude towards themselves. Feeling less shame was described as important for self-disclosure in family relationships, leading to increased understanding and support from others. Simultaneously, unchanged stigmatizing surroundings were described to relate to challenges with eating patterns and weight after end of treatment.

**Conclusion:**

Our findings indicate that relational symmetry, by patients experienced as being met with recognition, compassionate acceptance, and mutual investigation of subjective experience, can contribute to reduction of weight stigma and shame, and the burdensome notion of inferiority experienced by the participants in everyday life, hence improving treatment outcomes.

*Trail registration* The study was approved and registered by the Data Access Committee at Nord-Trøndelag Hospital Trust August 8th, 2019, registration number 2019_2335.

## Abstract (plain language summary)

Ten patients were interviewed about their experiences with participation in a 10-weeks weight- neutral treatment program for people with binge eating disorder (BED) and higher weight. The treatment given was group based, addressing stigma and shame, using models of attachment and affect regulation in the presentation of BED. Participants described a notion of inferiority towards other people due to weight stigma that maintained their attempts of dieting and weight loss with subsequent bingeing and weight regain. Participants and health care professionals’ mutual acknowledgement of driving elements of binge eating in treatment appeared to support participants feeling equal. Feeling less shame was described as important for self-disclosure, leading to increased understanding and support in relationships. Simultaneously, unchanged stigmatizing surroundings was described to relate to challenges with eating patterns and weight after end of treatment.

## Background

The burden of weight bias, and how it contributes to increased morbidity and mortality among people living with higher weight is well documented [1–3]. Weight-based stigma grows from an assumption that causes of obesity^1^ are under the individual’s control, and that obesity is a result of personal failure [4]. Based on this belief, people living with higher weight are characterized in negative wording by others in multiple situations, and more frequent exposure to stigma is reported to be related to increasing attempts to cope (with uncertain influence on emotional adjustment) and higher BMI [5].

A subgroup of individuals living with higher weight suffer from binge eating disorder (BED). Weight is not a diagnostic criterion in BED [6], and patients with BED therefore need not be of higher weight, however obesity and BED are strongly associated [7]. BED is defined by recurrent episodes of eating large amounts of food while experiencing loss of control, feeling distressed, along with the absence of inappropriate weight compensatory behaviors [6]. Lifetime diagnosis of BED is reported by 0.6–1.8% of woman and 0.3–0.7% of men worldwide [8]. BED-diagnoses were introduced in the 1950s but have not until recently been recognized as an eating-disorder category of its own [9]. Weight bias might have contributed to BED often being invisible and overlooked [8, 10]. Considering the etiology, stigma is a possible contributor to the development of BED as stigmatized people tend to adopt or internalize weight-bias as a way of thinking about themselves, leading to low self-esteem, evoking negative feelings, trigging binge eating behavior [11, 12].

Knowledge regarding stigma and how it might drive the development of both higher weight and BED should be integrated into clinical situations [4, 13]. To improve treatment and outcomes, a deeper understanding of how stigma and shame are related in healthcare practices is needed [14]. Qualitative research methods are well suited for investigating factors that may facilitate or hinder the effectiveness of interventions concerning people of higher weight [15]. Qualitative research on patients’ lived experiences from behavioral weight loss therapy are scarce compared to quantitative research, but findings indicate that treatment by healthcare providers include several negative experiences including lack of respect and compassion, verbal insults, inappropriate humor, unmet healthcare needs, and breaches of dignity [16]. In contrast, the significance of compassion, understanding, dignity and the right to be treated as individuals have by patients been stressed as important for a positive patient-health provider relationship [16]. Qualitative studies on patients’ experiences with BED treatment are limited, but Salvia and colleagues [17] found that patients having participated in a weight-neutral treatment program  reported mainly positive experiences, emphasizing the ability to self-advocate [17]. Still, a further investigation of  contributing factors to the promotion and support of self-advocacy skills is needed according to the authors [17]. Taken together, there is an overall need for a better understanding of patient experiences from BED treatment in higher weight populations.

The screening and treatment of BED in Norway is unsystematic and scarce, both in mental health care settings and specialized obesity-clinics. Therefore, a pilot study called People Need People (PNP) was designed [18]. PNP is a weight-neutral, group based psychoeducational treatment program emphasizing stigma and shame, using models of attachment and affect regulation in the presentation of BED [18]. The aim of the ten-week program is to expand the patients’ understanding of the driving elements of binge eating disorder, hence methods to reduce symptoms (such as weighing, exercise and diet recommendation, self-monitoring, and behavior reporting) are not included. Results regarding feasibility, symptom severity and change in binge-eating frequency and health related quality have earlier been reported [18]. The current study was designed to gain insight into how participating was experienced by patients. The research question was: What are the experiences of participating in a weight-neutral eating disorder treatment that specifically addresses the underlying factors contributing to binge eating disorder?

## Theoretical framework

The research was anchored in a phenomenological framework. In this perspective, individuals and their surroundings are intertwined, irreducible and dependent. Hence freedom is not regarded as absolute, but characterized by the specific situation or context the individual is in [19]. This implies that changes to either the environment or the individual somehow will affect the other.

The feminist phenomenologists Käll and Zeiler characterize this as the *Bodily, Relational Autonomy*, and state that individual capability for freedom or choice, is deeply rooted in the connectedness to others [20]. Anchored in this perspective, experiences related to the interaction between the participants and their surroundings, both in their everyday life and in the treatment-setting, are given specific attention in the study.

## Methods

### Study context, recruitment, and participants

Patients were referred from a specialized obesity out-patient unit in a tertiary care hospital to a mental health out-patient clinic for eating disorder treatment using the PNP-model.^2^ Patients underwent clinical assessments according to standard clinical psychiatric care, considering both eating disorder pathology and struggles with body image. A detailed description of participants, screening, and treatment is earlier published [18]. For the study presented in this paper, ten of fifty participants were recruited during a period from July to December 2020, in line with principals of purposive sampling in qualitative research [21]. Besides having participated in and finished the PNP-intervention, the sample was recruited with variation in demographic characteristics such as age, level of education, marital- and employment status (Table 1). Sufficient information power was determined according to items described by Malterud, Siersma and Guassora; study aim, sample specificity, use of established theory, quality of dialogue, and analysis strategy [22]. Common characteristics were high eating disorder pathology, BED-diagnosis, previous psychiatric treatment in psychiatric care for other mental illnesses, and higher weight bodies. Eight of the recruited participants had early onset experiences of preoccupation with body and food. All participants reported one or more adverse childhood experiences in line with the definition described by Felitti and co-workers [23]. All ten had participated in and completed the treatment program at the time of the interviews. TTEN was responsible for conducting the treatment sessions the patients took part in, and KHB was a co-therapist in the first of the five treatment groups conducted.

**Table 1:** Demographic and characteristics of the participants

Characteristics	Count
Gender	
Female	8
Male	2
Ethnicity	
White	10
Age	
20–30	2
30–40	3
40–50	3
50–60	2
Cohabiting status	
Living alone	4
Living with partner and/or children	6
Education level obtained	
Middle school	1
High school	5
University degree (minimum bachelor)	4
Employment status	
Full time employed	6
Student	1
Public welfare	3
Fulfilling DSM-V criteria for binge eating disorder	10
Earlier treatment for other diagnosis in specialized psychiatric outpatient care	10
Self-reported onset of preoccupation with body and/or food	
Pre-teen	4
Teens	4
Adulthood	2
Having experienced Adverse Childhood Experiences [23]	8

Eating disorder pathology^a^	Mean (SD)
EDE-Q global	4,62 (0,9)
CIA	34,1 (6,9)
Internalized shame scale	79,5 (9,7)

^a^Measures were given before treatment and is included in the table to illustrate levels of eating disorder pathology at admission

### Data generating

The study has a qualitative design. To capture the participants’ lived experiences, first and third authors conducted semi-structured interviews. The interviews took place, face to face, at the mental health clinic, each interview was audio-recorded and lasted for 60–90 min. TTEN performed the interviews, KHB sat by, observing, taking notes and asked questions for clarification. An interview guide consisting of three main topics was used as a rough thematic framing of the interviews. The participants were first asked to give descriptions of their everyday life, function, eating-disorder symptoms, and experiences from clinical settings prior to the intervention at hand (PNP). Secondly, participants were asked to share their experiences with the treatment’s content and form. Lastly, they were asked about their needs after finishing the treatment. Consistent with phenomenological informed interviewing, we encouraged the participants to elaborate on details regarding concrete experiences instead of sharing attitudes, opinions or interpretations related to situations [24]. We also aimed at being flexible and open to the way each participant narrated their experiences and the evolving of each interview [24].

### Data analysis

The interviews were transcribed verbatim by the first author and analyzed guided by van Manen’s hermeneutic-phenomenological approach [24]. All three authors performed a wholistic reading by reading all ten transcripts. Taken together, having experienced something distinct from earlier treatment stood out as significant. The first author then went on with a reading guided by the lens of the five existentials (lived body, lived time, lived space, lived relation, lived objects). Organization of the material through the existentials was presented for the second and third author, and relevance towards the main theme “having experienced something distinct” was discussed. The first author then wrote forth the analysis, now organized in three themes with the heading’s *inferiority*, *equality,* and *time capsule,* with the first theme overarching the two latter. In the final part of the analyses, first author integrated paragraphs from the interviews that exemplified and nuanced the three themes, re-wrote, and discussed with the co-authors.

The last readings were also partly driven by theory, or what van Manen describes as insight cultivators [24]. The concept *chronic shame* [25] guided the reading of experiences concerning self-perception and agency both within and outside clinical encounters. The concept *intersubjective thirdness* [26] was specifically fruitful when seeking to grasp the participants’ relational experiences from the treatment.

## Researcher characteristics and reflexivity

Both KHB and TTEN have lengthy clinical experience from specialized eating disorders treatment and psychiatric care in general. Staying open to the participants’ lived experiences without seeing through glasses of classifications such as diagnosis or symptoms, or in terms of change and recovery as symptom-reduction was central given the first and third authors’ clinical experience with eating disorders.

By involving an experienced, phenomenological oriented qualitative researcher (EN) in the analytic process, we sought to challenge and critically explore KHB and TTENs taken for granted knowledge. EN is an experienced researcher within the field of obesity and had no clinical or academic connection to the PNP-program prior to her contribution in the analytic work on the material.

## Findings

The first theme, *Inferiority—under constant (de)valuation*, captured how experiences with their bodies, symptoms of eating disorder, and treatment prior to attending the treatment intervention were described by the participants. This theme is recognized as vital for how the participants experienced the BED treatment that was given, and how they encountered life after end of treatment. In this sense, the first theme frames the two latter: *Equivalence*—*shared human ground* and *Time capsule—life on hold.*

### Inferiority—under constant (de)valuation

The participants were unison in their description of the body being wrong in profound ways.^3^ They experienced the body first and foremost as too large, having always felt that way, also in periods of slenderness. The body was perceived as an obstacle due to size, and the participants experienced pain, heaviness, exhaustion, and sleep-deprivation as barriers for activity and movement. The participants also described bodily awareness as an experience of chaos, where being large, triggered feelings like distress, fear, anger, and despair. Taken together, the participants experienced the body’s shortcomings as existential, defining their very being, as described by Gail:

It is a feeling of being a failure, that there is something wrong with me, that I am destroyed.

The complicated and negative relationship towards their own body was not merely described as related to weight. The participants also stitched their early acknowledgement of the body’s exposure and vulnerability to traumatic experiences such as severe violence, sexual abuse, bullying, and fear of alcoholic parents or partners. Commonalities in these experiences were the notion of an imbalance of power between themselves and others maintained by violence, or the threat of violence, leaving the participants exposed and vigilant and with a feeling of defenselessness as described by Ruth:

I have been vigilant since I was a little girl, I remember a bed in the house I grew up in, you could pull it out to be longer, I get sick when I see beds like that …. It was a sexual assault.

In addition to the negative perception of and thoughts about their bodies, experiences with weight stigma in professional life, among friends and within the family, were common for all participants. A general experience was that mothers had induced diets for them from an early age, or that mothers through comments, gazes or tactile communication woke their awareness of body-shape and weight. Beth said:

I have listened a lot to my mother, she also has an eating-disorder. We grew up with comments like “Do you really need that extra potato?” or “Do you really need that extra portion? It was always in my thoughts when eating.

Participants’ feelings of inadequacy compared to health-care professionals was described as a feeling of inferiority and vividly described by all participants. Clinical encounters were reduced to being screened and treated for different body parts not working because of their weight, according to the participants. The participants longed for health-care professionals to take interest in how living with a large body felt, and how the inability to lose weight or sustain weight loss affected their psychological wellbeing. They also described prior situations in health-care settings where they felt that their knowledge and experience was not considered, triggering a sense of humiliation and shame, a form of infantilization, as Eva formulated:

As a nurse in my late twenties, having three kids, I know some things about nutrition, I know what I should and should not eat, but there [in lifestyle-treatment] I found myself in a grocery store, with a group of obese people led by a skinny nutritionist, and we were told to look at the content declaration of different foods, a box of cottage cheese for example, it was so humiliating […] I guess the intentions were good, but the method, it was so shameful, she was young, fit and skinny, it was so obvious that it was “her and us”.

Being preoccupied with the bodies of professionals they had met in clinical encounters was furthermore verbalized. Slender bodies of the health care-workers made participants feel a marked distinction, placing themselves on the lowest shelves in the hierarchy of success and self-control. Social events with friends were also colored with the same self-consciousness and were often associated with extensive avoidance of social involvement. When socializing, the participants used rather ambivalent strategies to cope with the feeling of inferiority. On the one hand they described reluctancy to talk about their problems with the body (related to the eating-disorder and trauma), while on the other they described using humor, mocking their own body as a way of coping. Making fun of themselves created emotional distance, described by Beth:

I always made jokes about myself first, which may look like body-positivism to others, they don’t think that a fat person jokes about being fat, but in 99 % of incidents you will, because it hurts less if you joke about it first, before they do, because you know they will.

The only situation where the participants described the body in a positive way was when talking about planning and practicing binge eating. Binge eating started early in life for many of the participants. Phrases like *experiencing pleasure*, *being calm*, *reducing stress*, *my only joy*, *something to look forward to*, and *keeping all the pain on a distance,* were described as effects of binge eating episodes. However, the effect and the positive feelings prior or following binge eating episodes lasted for a short period of time, soon followed by diametrical sensations like pain, nausea, sweating, increased heart rate, along with feelings of anguish, disgust, shame, and being a failure. Binge eating was thus described as a deceitful way of relief. The painful aftermath often fueled the next binge eating episode, keeping the participants in a cycle of dieting, loss of control, bingeing, and a profound feeling of not coping with eating and weight loss.

Enhanced well-being and practical functioning in their everyday life was put forward as incentives for weight loss among the participants. However, diminishing extreme body-shame was described as the strongest motivation when entering therapy. A broad spectrum of diets and weight cycling over the years, both within and outside health-care settings, were described. The participants expressed regret for spending money on diets, and time lost dieting, but not achieving their weight-goal, however they kept on striving. Jane said:

I wanted to become thin, I just wanted to be thin, everyone that is thin are happy. I thought that if I became thin, everything would be just fine.

Feeling inferior to others due to the body being too large was central in the participants’ description of self-perception and everyday functioning throughout life. The body stood fourth as an object under constant surveillance and valuation, never found good enough, neither in the eye of the participant nor in the imagined eye of other people. Layer upon layer of embodied stigmatizing situations were unraveled, some colored with derogatory wording, others by violence. Words witnessing deep contempt for themselves were used by participants, and constant preoccupation with shame was expressed. Avoidance of, and in, social settings and binge eating behaviors were described as coping mechanisms for protection, stress reduction and to create positive emotions. The body was perceived as a constant reminder of inferiority among the participants, and dieting to become thin was expressed as a strategy to reduce feelings of relational asymmetry.

### Equality—shared human ground

The participants revealed that they had low expectations prior to the BED treatment. Most described an initial skepticism towards group-therapy, which was rooted in fears of being inhibited by social anxiety. Uncertainty and fear related to sharing experiences in general, and that their experiences would not be familiar or understandable by others were common among participants. The feeling of validation in therapy seemed somewhat like a surprise. Jane said:

Someone has understood what I say, it can’t be that wrong when numerus people are describing the same experiences, it can’t.

The underlying assumption of having false or wrong experiences (that trauma memory, shame, and loss of control over eating was connected), was challenged when their experiences were mirrored by fellow participants. Shared experiences were most often ones they had never verbalized. Kai said:

I feel like, there was a room (the therapy room) where I could talk, just a little bit, about my stuff, I was allowed to open on a subject I don’t talk to many others about, we trusted each other in the group. What the others talked about made sense to me. It made me look forward to coming. I did not worry.

The feeling of being validated occurred both when interacting with fellow patients, and when listening to and discussing the content of the sessions with the group therapists. The participants expressed that the topics complemented rather than contrasted their own experiences. Instead of pointing on certain themes that were important in the intervention, participants emphasized the totality of the treatment program. Eva summed it up:

I feel like, if you take the ten group sessions, it’s like they are ten missing pieces in a puzzle.

The structural and pedagogical foundations of the PNP intervention were described as pleasing to the participants. The therapists’ usage of everyday language, PowerPoints with little text, animated films to illustrate complex emotions, concrete examples to deepen theoretical points, and the invitation to use the paper and crayons spread were all described as important and useful approaches. The participants connected the pedagogical methods to feelings of being focused, giving them the ability to grasp the content of the sessions. Beth phrased the experience like this:

I felt very comfortable, I had no problems following what was presented, my thoughts were not drifting at all, I felt very focused.

The relationship with the group therapists was described with nuances altering the participants’ self-perception. The overall experience was described as a feeling of being on the same level, Jane said:

There has been no top-down attitude, I haven’t felt like a patient.

The participants made a distinction between being a patient and feeling like a patient, emphasizing the group therapists’ self-disclosures and usage of own examples showing vulnerability, and own shame experiences. The participants described that the therapists use of self-disclosure offered a sense of being of the same kind, that there was no “*us and them*”. Ann expressed:

They are not different, they are human beings, very human human beings.

The feeling of being met with an open mind and having the treatment-focus adjusted in line with what patients shared, was also held as important to the participants. The group sessions were experienced as open conversations, as opposed to a prefabricated lecture. Mutual turn-taking between health-care professionals and patients contributed to this experience. Gail said:

She [the group therapist] is curious of how I feel and think instead of telling me what to do.

Simultaneously, participants described therapists taking charge at crucial moments during sessions as essential for the therapists’ credibility, as a need of balance between being given the opportunity to think for themselves and being able to rest in the group-therapists responsibility of the therapeutic process was identified. For example, the participants liked that the therapists did not humor them and appreciated being challenged and asked to elaborate and clarify. They described consequently being stopped when talking themselves down, such as making jokes about themselves or expressing weight stigmatizing attitudes using words like lazy, stupid, or lacking discipline. Ruth stated:

She didn’t just echo me or the others, I can’t get away by saying that’s just the way it is, I can’t hide.

Perception of selfhood and agency was challenged and nuanced when interacting with fellow patients and health-care professionals in the treatment intervention. The experience of being equal stood forth as crucial to the participants. The importance of equality was especially valuable as it contrasted their past and repeated and profound feelings of inferiority. The participants described how structural and relational aspects of the treatment altered how they perceived distribution of power between themselves and others. Validation of the complexity of weight and life, and not being given a recipe to change, was central to their experiences.

### Time capsule—life on hold

Participants loss of hope for a better life was strongly connected to the passing of time without experiencing a substantial or desired change in neither everyday functioning nor weight, even in the hands of specialized healthcare.

A common experience among the participants was difficulties gaining access to mental health treatment despite being referred by their general practitioner with substantial mental health distress. Their binge eating disorder went undetected even after earlier psychiatric assessments, despite their disclosures of long-standing patterns of using food to regulate their emotions. Instead, participants described being labeled with a broad spectrum of other mental health diagnosis; mood-disorders, different anxiety-diagnosis, and personality disorders. However, some of the participants expressed that receiving a psychiatric diagnosis other than BED validated the multifaceted nature of their challenges, acknowledging the complexity of their struggles with food and weight. Even so, most felt left with unease, as central aspects of their suffering were ignored when assessed. Several expressed a feeling of failure when neither weight treatment in the obesity-clinic nor psychiatric health care helped them cope with their struggles with food, weight, nor body image. Ann described:

I was not examined […] I don’t know what they are doing, it seems like they just refer me away all the time […] you lose hope, all hope, no one can help, they just send you in circles.

Taking part in a weight-neutral treatment like PNP was described as entering a time capsule, yet a different one. Many described the treatment as validating and power-balancing. However, there also seemed to be a tension between the positive experiences with the treatment, and the limited endurance of the program as the participants experienced a strong need for continuation of the therapy, needing both psychotherapy and body-oriented follow-up treatment. The difficulty of being cut off from a therapeutic process they perceived as uncompleted was hard, and expressed like this by Sue:

I need help to put all the things I’ve learned into a system, learn how to continue to get out of it in the best possible way, I feel like everything is up in the air, but so far it just hangs there, I don’t know how to take it down.

Nevertheless, the participants ascribed meaningful and positive changes, such as tolerating and containing feelings, being able to differentiate between hunger and craving for binge eating due to distress, and a deeper understanding of themselves which led to a kinder and more caring attitude after finishing the PNP intervention. Moreover, feeling less ashamed was vital for participants, making openness and increased understanding and support in close relationships easier. However, worries of living in a society that stigmatizes larger weight bodies and the equality felt in the “PNP time capsule” was expressed as a discrepancy and Eva said:

We have used all our lives tearing ourselves down in a way, and ten therapy sessions is maybe not enough to repair that, now someone finally understood, and then we were let free again, it feels so short [crying] you want to stay longer. We go back to our lives where things are like they always were, people understand precisely as little as before, it is the same shame and stigma out there, it is not as protected and safe as in this [therapy] room.

The participants described gaining new experiences, not being judged, or stigmatized, while in therapy, however their surroundings remained the same. It appeared as if the participants felt the time stood still due to structural forces outside their control. The “time capsule” described was maintained by stigma, dieting as attempts to lose weight, and the notion of inferiority on constant repeat.

## Discussion

In this study exploring experiences with weight-neutral treatment for patients with binge eating disorder and higher weight bodies, participants described their relations to others interwoven with their eating disorder patterns. More specific, the profound feeling of inferiority due to weight stigma and adverse childhood experiences, kept the participants stuck in a shame driven carousel of dieting, weight loss, binging and weight regain. Inferiority was also associated with a spectrum of avoidance behaviors. Further, findings indicated that participants and health care professionals’ mutual acknowledgement of driving elements of binge eating, and subsequent weight increase, were described to lead to a feeling of equality, valuable for recovery through change in self-perception and agency. Simultaneously, unchanged stigmatizing surroundings were  described to relate to challenges with eating patterns and weight after end of treatment. The discussion will elaborate on dynamics of inferiority and equality and investigate implications for future treatment of patients with binge eating disorder and higher weight.

Inferiority and avoidance fueled by shame seemed to be an existential set off for the patients when signing up for treatment. Description of weight bias and adverse childhood experiences (ACEs) found in our study corresponds with research showing that people with higher weight and BED are prone to both systematic oppression through stigma [27] and high incident of ACEs (like abuse or neglect) [28, 29]. The body, through being the target for weight bias and maltreatment, was perceived as a constant reminder of inferiority among the participants, and dieting to become thin was expressed as a strategy to reduce shame and feelings of relational asymmetry. In addition, feeling inferior was described as causing various forms of avoidance behaviors affecting participants’ everyday life as their social involvement and willingness to self-disclose decreased. For example, avoidance of social events and ridiculing their own bodies when socializing with family and friends, were described as strategies to cope with *expectations of shame*. Shame grown out of time-limited, discrete events can be labeled chronic [25]. According to Dolezal, anticipation of shame can become a defining feature of the lived experience, causing a continuous sense of social anxiety, personal inadequacy, and relational disconnection [25], as found in our interviews. The avoidance behaviors described by the participants are also in line with empirical research on eating disorders. The binge eating behavior itself can be seen as an avoidance strategy of negative self-evaluations [12]. In addition, factors like triggers for eating, social eating situations, size- and shape-related information, public bodily exposure, intimate relationships, therapeutic programs, and social interacting, have been identified to stimulate avoidance behaviors [30]. Taking both our findings and previous research into account, shame driven avoidance strategies seems common among people suffering from BED, consequently it should be considered when planning and conducting treatment.

Participants experienced that their habitual avoidance to cope with inferiority was challenged when they engaged in the PNP-intervention. Participants described the opportunity to tell their story, feeling understood and mirrored, both by the content of the PNP and the way therapists delivered the treatment, reduced feelings of shame and stimulated feelings of equality. Valuation of the interpersonal relationship with clinicians and their ability to create a safe and comforting environment to promote change, corresponds to qualitative research on patients’ experiences with BED-treatment [31]. On the other hand, negative experiences with healthcare providers can be associated with greater levels of unmet healthcare needs [16, 17], as described by the participants in numerus treatment situations prior to the PNP*.*

Attributing change to qualities of the therapeutic relationship parallels with previous therapy- research [32], stressing the importance of alliance, collaboration, goal consensus, empathy, positive regard in addition to affirmation and collecting and delivering client feedback [32, p 309]. In addition, Benjamin argues that patients preserving subjectivity instead of feeling like an object for therapists’ observation and advice, and patients and therapists’ mutual recognition of each other’s subjectivity can buffer against feelings of shame and self-blame [26], as found in our study. That the participants associated reduced shame and feelings of equality with qualities of the therapeutic relation, is also in line with earlier suggestions on how to prevent reproduction of stigma in clinical settings for people with higher weight: educating health care professionals on the complexity of obesity and conducting weight inclusive approaches [4] and performing respectful treatment and secure access to quality health care [13].

Participants also experienced that reduced shame increased their awareness and tolerance of bodily sensations and emotions, and a deeper understanding and self-caring attitude towards themselves were expressed after finishing the treatment. Furthermore, they reported feeling less shame in relation to their loved ones, important for their self-disclosure and increased understanding and support in relationships. The positive changes correspond to effects compassion-focused therapy can have on ED symptoms [33] and body weight shame [34]. For example, do Carter et al. [34] stress the importance of patients’ improving the capacity to receive compassion from others, due to the insufficiency of relying solely on the self’s ability to reduce suffering. Associating a patient-centered therapeutic attitude with improved self-care was also reported by patients participating in weight-neutral BED treatment [17], who experienced that collaborative decision-making and goal setting (together with shift in therapeutic focus) impacted outcomes like symptom reduction, ability to self-disclose, improved self-compassion, and increased body acceptance impacted. Taken together, behavioral choices seem relative to perceived degrees of relational balance to others (e.g., the therapist, fellow participants, and loved ones). Therefore, the quality of relational connectedness [20] between the participants and health care professionals seems to be of high relevance to treatment-outcome. Understanding the stigma, shame, and avoidance triad, by participants perceived as inferiority, may support health care providers in creating a social environment characterized by equality between the parties. This might be a path to improve quality of treatment for patients with BED and higher weight.

In addition to context dependent feelings of inferiority and equality, a third core finding relevant for treatment outcome was discrepancy between a safe environment in therapy and perceived weight stigma in everyday life. Participants expressed fear that weight stigma outside the treatment setting would corrupt feelings of equality experienced in the PNP-intervention, and that their improved function would not sustain after treatment. Maintenance of dieting, bingeing and subsequent weight gain in stigmatizing surroundings is understandable, as our findings indicate that attempts at weight reduction often is induced to compensate for feelings of inferiority related to weight stigma. Earlier research signals, like our findings, that participants in weight-neutral treatment might hesitate to give up dieting due to strong cultural and societal factors [17]. Socio-political aspects of chronic shame and BED such as oppression, domination, exclusion, and marginalization, have also been addressed by Dolezal [25] and Keski-Rahkonen [8] respectively, possibly hindering effective treatment [8, 14]. Barnes and colleagues [35, p 180] argue that *pervasive stigma among both providers and patients could be a potential threat to treatment.* This finding corresponds to research documenting that internalizing negative societal or cultural beliefs about own body weight can have negative implications for healthcare [36].

Despite these barriers to successful treatment of binge eating disorder, we argue in favor of weight-neutral approaches. Aiming at facilitating patients’ ability to be self-compassionate and practice self-advocacy, weight neutral treatment can stimulate patients with BED and higher weight to gain a more balanced eating pattern, contributing to improved psychosocial wellbeing and healthier biomarker profiles [17, 37], instead of perusing dieting as adjustment to oppression.

### Strengths and limitations

Considering the damaging effects of weight stigma in clinical encounters, inclusion of patient experiences with BED in development and evaluation of treatments is needed. Hence, conducting in-depth interviews with participants to investigate subjective experiences from the PNP-intervention, can be regarded as a methodologic strength of this study. Participants had a wide variety of demographic characteristics and BED diagnosis were confirmed by clinicians using diagnostic manuals, thus contributing to transferability of findings. Furthermore, two of the researchers’ familiarity with the patient population could be valued as a strength in this study as clinical experience generated a rich and nuanced material. However, the first and third author’s two-fold roles towards the participants might be considered a limitation, with a need for reflexivity [38, 39]. Participants may have constrained their potential sharing of difficult or shame-evoking experiences from interactions with us in the treatment setting when being interviewed as participants in the study. First, because assertiveness is a core challenge for people suffering from eating disorders [40]. Second, it might have been perceived crucial to the participants to maintain a good relationship with us as health-care professionals when treatment-alternatives for patients with BED are limited or non-existent in Norway. Hence, fear of compromising their future treatment of eating disorders might have restricted the participants in sharing experiences that could be recognized as implicit critique by us, or the treatment given. Consequently, we might have missed nuances in the informants’ experiences that could have enriched our analysis, possibly verbalized with researchers they had had no prior relation to. However, establishing a relationship with the participants during the PNP-intervention, identifying driving factors of their binge eating, might have lowered barriers that often inhibit people with higher weight to talk about shameful experiences, also reported by the participants. Finally, when analyzing the material, first and last authors familiarity with BED might have limited openness when interpretating the data. Letting theoretical insights partly guide the analysis, involving an external reader (second author), performing thick descriptions of methodological dispositions aiming to be transparent and reflexive, were efforts made to communicate validity in our study [41, 42].

## Conclusion

In this study we found that a profound feeling of inferiority due to weight stigma appeared to have kept the participants stuck in a shame driven behavioral cycle of dieting, weight loss, bingeing and weight regain. Participants emphasized the mutual investigation and understanding of binge eating disorder between patients and therapists, and it appeared to stimulate a feeling of equality. The findings underpin the importance of establishing good relational dynamics between patients and therapists in BED treatment. Further, feeling less shame during treatment was described as encouraging self-care, increasing self-disclosure and subsequent understanding and support from others, altogether important for their recovery. However, unchanged stigmatizing surroundings was described to relate to challenges with eating patterns and weight after end of treatment.

Our findings indicate that relational symmetry, by patients experienced as being met with recognition, compassionate acceptance, and mutual investigation of subjective experience, can contribute to reduction of weight stigma and shame, and the burdensome notion of inferiority experienced by the participants in everyday life, hence improving treatment outcomes.

The positive experiences from participating in this weight-neutral eating disorder treatment specifically addressing the underlying factors contributing to binge eating disorder calls for further investigation. Future research to provide a better understanding of weight stigma in treatment settings and impact of internalized weight stigma on treatment outcomes are needed. Efforts at reducing weight stigma at a systemic, societal, and cultural level is beyond the scope of treatment, but can be addressed in exploration of the etiology of BED.

## Data Availability

To ensure full anonymity for the participants, the data generated and analyzed during the current study are not publicly available. Transcribed interviews are available from the corresponding author on reasonable request*.*

## Abbreviations

ED: Eating disorder
BED: Binge eating disorder
PNP: People need people
EDE-Q: Eating disorder examination questionnaire (version 6.0)
CIA: Clinical impairment assessment questionnaire
ACE: Adverse childhood experience

## Footnotes

1 We are aware that language is important when dealing with complexity and conditions that are stigmatized. We use the term *obesity* because the paper is written for a medical audience, and patients are admitted to treatment due to being diagnosed with severe obesity. Talking with and about patients with higher weight we avoid the term obesity as research report people finding the term stigmatizing.

2 Patients that are admitted to the obesity-clinic have a BMI ≥ 40, or ≥ 35 with comorbid medical complications.

3 The participants described a polarized perception of the body. Sometimes the body was experienced in negative terms as described here, other times as silent or cut off from consciousness. This dual notion of the body can be interpreted as an expression of body-image distortions and will be investigated in depth in a separate paper.
